# Healthcare Expenditures and Reimbursement Patterns in Idiopathic Pulmonary Fibrosis: A 10-Year Single-Center Retrospective Cohort Study in Turkey

**DOI:** 10.3390/healthcare13172084

**Published:** 2025-08-22

**Authors:** Kerem Ensarioğlu, Berna Akıncı Özyürek, Metin Dinçer, Tuğçe Şahin Özdemirel, Hızır Ali Gümüşler

**Affiliations:** 1 Pulmonary Medicine Department, Faculty of Health Sciences Ankara Atatürk Sanatoryum Training and Research Hospital, Ankara 06610, Turkey; 2 Health Management Department, Faculty of Health Sciences, Ankara Yıldırım Beyazıt University, Ankara 06170, Turkey; 3 Invoice Department, Ankara Atatürk Sanatoryum Training and Research Hospital, Ankara 06610, Turkey

**Keywords:** antifibrotic agents, diagnostic tests, healthcare costs, idiopathic pulmonary fibrosis, nintedanib, pirfenidone

## Abstract

Background/Objectives: Idiopathic pulmonary fibrosis (IPF) is a chronic and progressive fibrosing interstitial disease that incurs significant healthcare costs due to diagnostic and treatment needs. This study aimed to estimate healthcare expenses related to IPF diagnosis, treatment, and follow-up, including factors affecting overall expenditure. Methods: This retrospective cohort study included 276 IPF patients from a tertiary hospital (2013–2022). Diagnostic and treatment costs were analyzed, including antifibrotic medications (pirfenidone and nintedanib), diagnostic tests (pulmonary function tests and performance evaluation tests), and interventions (fiberoptic bronchoscopy, imaging modalities). Costs in Turkish Lira were converted to United States dollars. Statistical analysis was performed using non-parametric tests to evaluate expenditure correlations with demographic, clinical, and treatment parameters, which included the Mann–Whitney and Spearman Rank Correlation tests when appropriate. Results: The median healthcare expenditure was USD 429.1 (9.13–21,024.57). Inpatient costs (USD 582.67; USD 250.22 to USD 1751, 25th and 75th percentile, respectively) were higher than outpatient costs (USD 192.36; USD 85.75 to USD 407.47, 25th and 75th percentile, respectively). Antifibrotic regimens did not differ significantly in cost or duration (Z = 0.657; *p* = 0.511) (mean pirfenidone duration: 1.1 ± 1.0 years; mean nintedanib duration: 0.6 ± 0.9 years). Diagnostic tests, particularly pulmonary function tests (PFT) (*p*: 0.001, Rho: 0.337), diffusing capacity of the lungs for carbon monoxide (DLCO) (*p*: 0.001, Rho: 0.516), and high-resolution computed tomography (HRCT) (*p*: 0.001, Rho: 0.327), were the primary drivers of costs. Longer treatment duration was positively correlated with expenditure (Rho: 0.264, *p*: 0.001 and Rho: 0.247, *p*: 0.006 for pirfenidone and nintedanib, respectively) while age showed a weak negative correlation (Rho = −0.184, *p* = 0.002). Gender and type of antifibrotic regimen did not show any significant effect on costs. Discussion: Diagnostic and follow-up testing were the main contributors to costs, driven by reimbursement requirements and the progressive nature of IPF. Antifibrotic medications, although expensive, provided clinical stability, potentially reducing hospitalization needs but increasing long-term care expenses. Variations in healthcare systems affect expenditures, with Turkey’s universal coverage lowering costs compared to Western countries. The study’s main limitations include being a single-center, retrospective study and its inability to include comorbidities and disease severity in the statistical analysis. Conclusions: IPF management is resource-intensive, with diagnostic tests and follow-up driving costs independent of demographics and treatment modality. Anticipating higher expenditures with prolonged survival and evolving treatment options is crucial for healthcare budget planning. Preparation of healthcare policies accordingly to these observations, which must include an overall increase in cost due to treatment duration and survival, remains a crucial aspect of budget control.

## 1. Introduction

Idiopathic pulmonary fibrosis (IPF) is a progressive, chronic form of fibrosing interstitial pneumonia with unknown cause and a median survival of 2.5 to 4.5 years. It is commonly seen in the elderly population and is characterized by pulmonary parenchymal fibrosis [1]. It is the most common type of idiopathic interstitial pneumonia and also the most severe in terms of clinical presentation. The incidence of IPF increases with age, and approximately 3 million people worldwide are affected [2]. With the aging population and new diagnostic techniques, the disease has been recognized more often, with a reported prevalence of 495 out of 100,000 elderly in the United States [3].

The pulmonary function test (PFT) often remains stable during the early stages of the disease, while the restrictive pattern is predominantly in the late stages. Reduction in total lung capacity, functional residual capacity, residual volume, and diffusing capacity of the lung for carbon monoxide (DLCO) is often observed. High-resolution chest tomography (HRCT) remains the mainstay imaging modality for IPF diagnosis, and the usual interstitial pneumonia pattern is typical of IPF. Invasive diagnostic procedures include bronchoalveolar lavage, transbronchial biopsy, transbronchial cryo-biopsy, transthoracic biopsy, endobronchial ultrasonography-assisted transbronchial biopsy, video-assisted thoracoscopic surgery, and surgical biopsy. The follow-up of the patients consists of clinical, functional (via a six-minute walking test and PFT), and morphological evaluation (by radiological imaging) [4].

IPF has been recognized as a healthcare burden due to its incidence, prevalence, and overall morbidity and mortality [5]. Among the insured population over 65 years old in the United States of America (USA), a rise in IPF diagnoses has been reported, supporting the idea that overall healthcare expenditure would increase [6]. Because managing patients with IPF requires extensive resources, the estimated annual cost per patient ranges from 29,000 to 37,000 United States Dollars (USD) [7].

Hospitalization costs have also been another significant item of healthcare expenditure for IPF patients, as overall hospital stays are usually lengthy, with additional treatment requirements during admission [8]. The available treatment options for IPF have been limited to lung transplantation as a definitive approach, alongside pharmacological and non-pharmacological options. Recently, antifibrotic drugs have been shown to slow disease progression by limiting the loss of lung function [9]. Two drugs, pirfenidone and nintedanib, have been approved for mild and moderate forms of IPF. These treatments also add to the cost, with an average monthly expense ranging from USD 2000 to 14,000 [10]

The burden of IPF and its associated costs to the healthcare system have recently garnered interest, with studies revealing unexpected expenditures due to overall longer survival, investigations related to screening and diagnostic procedures, and especially treatment-related costs [11,12]. Excessive costs have been reported in the USA, but studies examining the overall expenditure in universal healthcare settings, such as in Turkey, remain limited [13]. Considering Turkey’s diverse healthcare reimbursement system, where nearly all IPF-related healthcare costs are covered, different factors influencing expenditure may emerge. This could justify an extended healthcare coverage system for other countries or highlight areas of spending that may be unaffordable for countries with limited coverage.

This study aims to quantify the direct medical costs associated with IPF diagnosis, treatment, and follow-up under Turkey’s universal healthcare system, and to identify factors associated with higher expenditure. We hypothesize that diagnostic monitoring and prolonged antifibrotic use are primary cost drivers.

## 2. Materials and Methods

The study population included patients evaluated at a tertiary hospital specializing in pulmonary medicine from 2013 to 2022 in Ankara, Turkey. Patients diagnosed with interstitial lung disease (ILD) were included. Diagnostic history and medication reports for nintedanib and pirfenidone were obtained from the hospital system. The primary inclusion criterion was a confirmed diagnosis of idiopathic pulmonary fibrosis (IPF). A confirmed IPF diagnosis was based on American Thoracic Society and European Respiratory Society (ATS/ERS) guidelines at the time of diagnosis, which was available according to Turkey’s drug reimbursement policy that required an exact diagnosis and excluded other ILDs [14]. Patients with typical radiological findings of IPF, consisting mainly of subpleural and basal dominant involvement, presence of honeycombing, thickening of interlobular septa, and limited ground glass opacity, were diagnosed with IPF—after exclusion of other common causes of ILD, including rheumatological involvement. When radiological evaluation was inconclusive, tissue sampling was the preferred method for diagnostic confirmation, with video-assisted thoracoscopic sampling favored. Exclusion criteria included diagnosing patients at another center, regardless of follow-up duration there. Demographic data, pulmonary function tests, performance evaluation tests, and interventions like fiberoptic bronchoscopy, additional procedures, imaging methods, and total treatment duration were recorded. Performance evaluation tests encompassed any exercise test performed in a clinical setting, such as the six-minute walk test and the incremental shuttle test. Missing data in terms of testing evaluation were re-investigated from the patient file archive in the hospital, and in cases where these records were missing, patients were removed from the study. Due to retrospective nature of the study, no loss to follow-up was expected.

Total treatment duration was calculated based on how long a patient was on either nintedanib or pirfenidone. Any reason for stopping treatment, including adverse effects, treatment switch, or death, was considered the end of treatment for that particular drug, and the total duration was calculated accordingly. The Social Security Institution, which is the largest purchaser of health services in Turkey, was used to determine costs. Service fees were based on the Health Implementation Communiqué Prices issued by the Social Security Institution, which sets the tariffs for health services. Overall expenditure data was obtained from the hospital invoice department, where a two-step confirmation process was required for each patient—one on the hospital side confirming the bill and another on the Social Security Institution side confirming reimbursement.

All expenditure data were received in Turkish Lira and then converted to USD using the yearly exchange rate provided by the Central Bank of the Republic of Turkey’s archive. This rate was chosen based on the reimbursement rate of the Social Security Institution, which accepted a set USD value in accordance with policy, using the previous year’s annual exchange rate. This served as the state’s reference for reimbursement regarding overall healthcare, including nearly all expenses. Direct costs that could be billed included the overall cost definition, covering hospital admissions, outpatient evaluations, laboratory tests, medication costs, physician fees, and diagnostic imaging. Any other indirect expenditures were excluded. The study was conducted after receiving approval from the hospital ethics committee of Ankara Ataturk Sanatoryum Training and Research Hospital, with additional final ethics approval granted by the Ankara Yıldırım Beyazit University Ethics Committee for Health Sciences (Approval date 20 December 2022, approval number 2022-1316).

### Statistical Analysis

Parameters accepted as scale variables in the study were evaluated for distribution patterns using graphical analysis, the Kolmogorov–Smirnov test, and the Shapiro–Wilk test, all of which indicated that the variables were non-parametrically distributed. Descriptive analysis was conducted, and data were presented as means and standard deviations for easy comparison with other studies, along with median values and interquartile ranges for statistical clarity. For categorical variables (such as gender, treatment modalities, intervention history, presence of performance evaluation, and respiratory function tests), absolute counts and percentages with proportions were reported. Non-parametric tests were used due to significant differences in parameter ranges and non-standard distributions. To examine the effect of gender on total healthcare expenditure, the Mann–Whitney test was employed. The Spearman Rank Correlation test was performed to assess correlations between non-parametric variables. The statistical analyses were conducted using Microsoft Excel 2016 and IBM SPSS Statistics 22.0 (IBM Corp. Released 2013. IBM SPSS Statistics for Windows, Version 22.0. Armonk, NY, USA, IBM Corp.). A *p*-value below 0.05 was considered statistically significant.

## 3. Results

**Figure 1:**
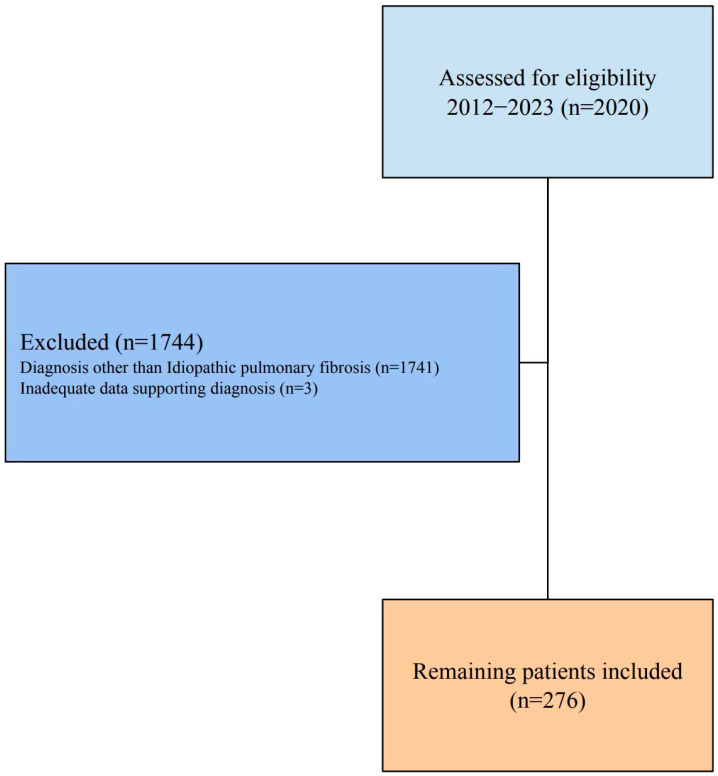
Patient Evaluation Flow Chart.

A total of 2020 patients diagnosed with ILD between 1 January 2012, and 1 January 2023, were evaluated for the study. Patients diagnosed with a different type of ILD other than idiopathic pulmonary fibrosis were excluded (n = 1741). The remaining 279 patients were investigated, and three were later excluded due to insufficient data confirming the diagnosis of IPF (Figure 1). The final patient population included 276 individuals, most of whom were male (n = 228, 82.8%). The average age was 66.0 ± 9.1 years, with a range from 34 to 87 years; age did not differ between genders (Z = 0.367; *p* = 0.713). The majority of patients being male and mean age being 66 years old were consistent with global IPF studies. All patients were on an antifibrotic regimen, with 196 (71.0%) patients on pirfenidone and 122 (44.2%) receiving nintedanib. The 48 patients with a treatment history of switches had used either nintedanib or pirfenidone at different times. The median healthcare expenditure was USD 420.09 (204.67–1188.61), with outpatient costs significantly lower than inpatient care, which had medians of USD 192.36 to USD 582.67 (Table 1).

The median duration for pirfenidone treatment was 1.1 years (0–5), while median treatment duration of nintedanib treatment was 0.6 years (0–4) (Table 2). Videothoracoscopy (n = 2, 0.7%) and computed tomography-assisted transbronchial biopsy (n = 1, 0.4%) were observed to be rare, and further statistical evaluation could not be performed for these results. Similarly, fiberoptic bronchoscopy (FOB) performed for stenosis treatment was reported to be three procedures (1.1%) and excluded from statistical evaluation. These observations were in line with the guidelines, which suggested these invasive options only for patients not diagnosed with other methods. Pulmonary function tests, including PFT, DLCO and HRCT were the most commonly performed tests (n = 207, 75%; n = 244, 84.4%; and n = 217, 78.6%, respectively). These testing modalities were high as expected, due to their predominant role in the initial diagnosis. Performance evaluation tests, including six-minute walking tests, were conducted on 80 (29.0%) patients. Positron emission tomography was requested for 26 (9.4%) patients. FOB and FOB with biopsy were performed on 27 (9.8%) and 16 (5.8%) patients, respectively (Table 2).

Pirfenidone and nintedanib regimens did not differ between genders (X^2^ = 2.046; *p* = 0.153 and X^2^ = 0.792; *p* = 0.354, respectively). Gender also did not influence the treatment switch (X^2^ = 0.319; *p* = 0.572). Similarly, other testing parameters did not vary by gender, including PFT, DLCO, HRCT, and FOB (*p* > 0.05). The lowest healthcare expenditure for IPF patients was USD 9.13, and the highest was USD 21,024.57. These variations in expenditure were assumed to either partial cases, in which only one component of the evaluation was possible. For the other end of the spectrum, extreme spending was mainly attributed to inpatient costs. The average cost of care was USD 1268.77 ± USD 2582.0, and the median expenditure was USD 429.1. Gender did not affect healthcare expenditure (Z = 0.064; *p* = 0.949). After excluding patients with treatment switches, the overall healthcare expenditure remained unaffected by the nintedanib or pirfenidone treatment regimens (Z = 0.657; *p* = 0.511) (Table 3).

Pirfenidone treatment duration showed a weak but positive correlation with PFT, DLCO, and HRCT test count (*p*: 0.001, Rho of 0.285, 0.278 and 0.261, respectively). Similarly, nintedanib treatment duration was positively correlated with PFT, DLCO, HRCT, and performance evaluation tests (*p*: 0.03, 0.001, 0.008; Rho of 0.305, 0.412 and 0.262, respectively). Overall, healthcare expenditure was positively associated with longer treatment duration, regardless of the treatment regimen. Performance evaluation tests were positively correlated with overall health expenditure (Rho = 0.264; *p* < 0.001), while no correlation was found between performance evaluation tests and other testing parameters (PFT, DLCO, and HRCT) (*p* > 0.05). DLCO was positively correlated with PFT and HRCT, with moderate strength (*p* < 0.001). A similar moderate correlation existed between DLCO and overall healthcare expenditure (Rho = 0.516; *p* < 0.001). The total count of DLCO tests is also reflected in the overall cost, with increased testing leading to higher expenditure (Table 3).

A negative but not statistically significant correlation was observed between age and treatment duration, performance evaluation tests, and PFT (*p* > 0.05). DLCO, HRCT, and overall healthcare expenditure showed a weak yet statistically significant negative correlation with age (*p* < 0.01). The overall healthcare expenditure had a weak positive correlation with PFT, DLCO, HRCT, performance evaluation tests, and treatment duration (*p* < 0.05). However, age exhibited a very weak negative correlation with healthcare expenditure (Rho = −0.184; *p* = 0.002) (Table 3).

## 4. Discussion

An increase in treatment duration was associated with higher overall care costs, as expected. Tests such as PFT, DLCO, HRCT, and performance evaluations also contributed to healthcare expenses. The negative correlation between age and expenditure may be explained by the more conservative approach to diagnosing elderly patients and their lower overall survival, which ultimately resulted in reduced compensation for IPF-related costs. Other potential reasons include under-treatment of these patients due to factors related to patient condition, which could limit medication use, or generally lower survival rates linked to age and possible comorbidities. Although IPF primarily affects males, gender did not influence expenditure or testing requirements. Similarly, the type of medication used did not impact overall costs. Overall, we can conclude that the main drivers of expenditure were the requested respiratory function tests rather than demographic factors or medication choices. Optimization of these tests, considering their role after initial drug costs, may contribute to reduction in healthcare expenditure; however, as stated in the age correlation, care should be given not to reduce tests to a point of underdiagnosis.

Protocols that are consistent with available guidelines, including national and international, such as ATS/ERS, should be utilized in optimization of tests, as even on a hospital basis, a standardized approach may be useful in cost reduction.

The choice of drug, such as pirfenidone and nintedanib, did not impact the overall treatment cost, showing similar effect sizes. This supports the idea that, regardless of the treatment method, the total cost remains stable as long as the patient is on an antifibrotic regimen. It also naturally suggests that drug expenses are a major component of overall healthcare costs, especially considering the reimbursement requirements for antifibrotic drugs, which involve at least one detailed evaluation every six months, including performance assessments and respiratory function tests. As mentioned, all pulmonary function tests and performance evaluations added to the total cost, and because they are necessary for drug reimbursement beyond routine assessments, the need for antifibrotics inadvertently increased overall costs due to secondary expenses. This lack of difference in drug choice might not apply in other settings, as studies comparing and reporting differences in drug regimens exist; therefore, a definitive statement regarding prescribing practices and drug selection cannot be made [15].

The hidden and relatively understated cost of drugs was mentioned in the study by Wong et al., where the expenditure was defined as one of the main expenses that could not be eliminated or reduced regardless of the country, with an undesirable cost-effectiveness profile [16]. An additional requirement and related expense of pulmonary and exercise function tests could be that these tests are also performed to evaluate the need for pulmonary rehabilitation. A longer follow-up period, which was expected from patients on an antifibrotic regimen, would eventually result in shorter evaluation periods with these tests due to the investigation of function loss for potential pulmonary rehabilitation needs.

The drug regimens used for IPF have been reported to improve overall survival, reduce flare-ups, and slow the decline in forced expiratory volume values. Although these benefits could decrease healthcare costs related to managing flare-ups and reduce the need for outpatient, emergency, and inpatient treatments, the increase in overall survival may also lead to higher total care costs. However, an exact cutoff point cannot be defined because, given the progressive nature of IPF, using a control group would not be feasible or ethical for cost comparison related to auxiliary expenses. The study by Kreuter et al. reported that medication accounted for nearly half of all expenses, with hospital admissions responsible for 40% of the total cost [17]. Another study by Nili et al. evaluated patients with progressive pulmonary fibrosis and found that overall costs were higher than those with non-progressive disease, mainly due to inpatient treatment requirements [18]. These studies support our assumption that medications are the primary drivers of expenditure, and longer survival inevitably results in more hospital admissions and additional healthcare costs during the disease course.

The overall cost of care was relatively lower compared to the literature, as a study reported a median cost of USD 32.834 in Western countries, with another study reporting a median Medicare cost of USD 20.887 [6,16]. This difference could be explained by currency exchange rates and variations in healthcare systems, as in Turkey, nearly all patients are covered by the Social Security Institution. This near-universal healthcare system covers all medical expenses, including antifibrotic treatment, IPF-related comorbidities, and hospital admissions.

Overall, it can be stated that, due to the significant bargaining power, which often results in lower payments to pharmaceutical companies based on currency exchange rates, it may have reduced the overall cost of care for IPF. This could potentially lower financial toxicity and out-of-pocket expenses for many patients. This conclusion might be supported by a study conducted in Germany, where the total cost was reported as € 8784, significantly less than in U.S.-based studies [17]. Insurance coverage remains a critical issue in European countries as well, as a study in Greece reported that half of the patients had coverage for IPF, while those without coverage faced out-of-pocket expenses for IPF-related medical needs [19].

The study did not find a cost difference between the two groups of antifibrotics, which was expected given the equal reimbursement status in Turkey and the fact that both drugs were approved for IPF treatment with no demonstrated superiority. However, in other countries, different findings were reported, with Cottin et al. describing nintedanib as more expensive in France [15]. A study from the USA noted that while the overall costs of the drug regimens were similar, the yearly drug cost for pirfenidone was lower compared to nintedanib [20]. The cost-effectiveness of the antifibrotic should also be considered from a disease management perspective. Supporting this role of antifibrotic treatment in controlling the disease, two studies in Spain suggest a general decrease in healthcare spending due to better disease control and fewer flare-ups under antifibrotic regimens [21,22].

The study had several limitations, either due to the study design or data evaluation related to reimbursement. The distribution of patients based on comorbidities, socioeconomic status, and disease progression were key parameters that could not be investigated. This was mainly because of the study’s time period, during which criteria for IPF progression changed multiple times, and because progression was not included as a separate parameter in the hospital patient database. Treatment duration with antifibrotics, along with treatment switch requirements, was used as an alternative method for disease evaluation, although it was not considered an exact measure. Socioeconomic data was only utilized in the reimbursement system up to 2008, in accordance with Turkey’s policy of providing a separate healthcare system for underprivileged individuals and those with low socioeconomic status. Eventually, this separation was removed to establish a nationwide healthcare system, which meant that socioeconomic status evaluations could not be performed on our patient group. Additionally, comorbidities and mortality assessments were not possible, as detailed comorbidity diagnoses could not be retrieved from the reimbursement system, which only provided treatment data and drug reimbursement information. Similarly, only overall mortality could be observed, as the exact cause of death was not available in terms of expenditure evaluation.

Another limitation of the study was the uneven distribution of hospitalization costs between inpatient and outpatient statuses. Consequently, even with an adequate model and an acceptable adjusted R^2^ value, it was not possible to make a clear division between inpatient and outpatient cost comparisons. Therefore, a regression analysis to evaluate the parameters affecting overall expenditure could not be developed. Similarly, the distribution pattern between the pirfenidone and nintedanib groups was non-parametric and varied widely; thus, only a non-parametric comparison could be performed.

## 5. Conclusions

IPF is a rare disease with limited treatment options and a high burden of diagnostic procedures. Most of the costs are related to treatment modalities, while diagnostic tests also contribute to overall expenses, regardless of demographics and treatment types. Follow-up duration is another factor affecting total costs, due to the repeated nature of evaluation tests. Although new guidelines recommend a clinical–radiological approach for diagnosing IPF, the expanding treatment options and longer patient survival and follow-up are expected to increase healthcare costs over time. Budget planning for IPF should account for these findings. Partial or bundled payments for specific testing methods, along with improvements in diagnostic techniques like HRCT, may help reduce costs for patients. Establishment of protocols for diagnostic approaches and adherence to those, on an international and national level, is also essential for cost management.

## Figures and Tables

**Table 1:** Demographic Characteristics, Treatment Modalities and Healthcare Expenditure in USD.

Parameters		n (%)
Gender	Male	228 (82.7)
	Female	48 (17.3)
Age (Years, ±SD)		66.0 (91)
Treatment Modality	Pirfenidone	196
	Nintedanib	80
Treatment Switch	Pirfenidone to Nintedanib	34 (12.3)
	Nintedanib to Pirfenidone	14 (5)
Healthcare Expenditure (USD, Median, 25–75th IQR)	Outpatient	192.36 (85.75–407.47)
	Inpatient	582.67 (250.22–1751)
	Total	429.09 (204.67–1188.61)

SD: Standard Deviation, IQR: Interquartile Range, Healthcare Expenditure definition included all direct costs, including interventions, imaging modalities, functional evaluation tests and treatment costs.

**Table 2:** Interventions and Diagnostic Methods.

Parameters	Performed (n, %)
Interventions	
Fiberoptic Bronchoscopy	27 (9.8)
Fiberoptic Bronchoscopy with Biopsy	16 (5.8)
Fiberoptic Bronchoscopy with Stenosis Treatment	3 (1.1)
Videothoracoscopy	2 (0.7)
CT-Assisted Transbronchial Biopsy	1 (0.4)
Imaging Modalities	
HRCT	217 (78.6)
Positron Emission Tomography	26 (9.4)
Functional Evaluation	
DLCO	244 (88.4)
Pulmonary Function Test	207 (75.0)
Performance Evaluation Test	80 (29.0)

CT: Computed Tomography, DLCO: Diffusing Capacity of the Lung for Carbon Monoxide, HRCT: High-Resolution Computed Tomography.

**Table 3:** Correlation between Parameters Regarding Overall Cost in USD.

Parameters		Overall Cost (USD)	Age	HRCT	DLCO	Pulmonary Function Test	Performance Evaluation Tests	Nintedanib Treatment Duration
DLCO	Rho	0.516	−0.18	0.457		0.462	0.088	0.412
	*p*	0.001	0.005	0.001		0.001	0.46	0.001
Pulmonary Function Test	Rho	0.337	−0.113	0.316	0.462		0.194	0.305
	*p*	0.001	0.104	0.001	0.001		0.11	0.003
HRCT	Rho	0.327	−0.182		0.457	0.316	0.073	0.262
	*p*	0.001	0.007		0.001	0.001	0.541	0.008
Performance Evaluation Tests	Rho	0.273	−0.05	0.073	0.088	0.194		0.344
	*p*	0.014	0.662	0.541	0.46	0.11		0.05
Pirfenidone Treatment Duration	Rho	0.264	−0.09	0.261	0.327	0.285	0.198	0.015
	*p*	0.001	0.209	0.001	0.001	0.001	0.124	0.921
Nintedanib Treatment Duration	Rho	0.247	−0.133	0.262	0.412	0.305	0.344	
	*p*	0.006	0.145	0.008	0.001	0.003	0.05	
Age	Rho	−0.184		−0.182	−0.18	−0.113	−0.05	−0.133
	*p*	0.002		0.007	0.005	0.104	0.662	0.145

Rho: Spearman rank correlation coefficient, DLCO: Diffusing Capacity of the Lung for Carbon Monoxide, HRCT: High-Resolution Computed Tomography; Performance Evaluation Test definition included the six-minute walking test and its variations.

## Data Availability

The original contributions presented in this study are not publicly available as per Turkey’s Personal Data Protective Authority law, and is available from authors upon reasonable request and approval from the mentioned Institutional Review Board.

## Abbreviations

The following abbreviations are used in this manuscript:

IPF	Idiopathic Pulmonary Fibrosis
ATS	American Thoracic Society
ERS	European Respiratory Society
USA	United States of America
PFT	Pulmonary Function Test
DLCO	Diffusing Capacity of the Lung for Carbon Monoxide
HRCT	High-Resolution Chest Tomography
ILD	Interstitial Lung Disease
USD	United States Dollar
RHO	Spearman’s Rank Correlation Coefficient
